# Diagnosis and Surgical Treatment of Suppurative Pelvic Diseases1Read before the Buffalo Academy of Medicine, June 28, 1898.

**Published:** 1898-11

**Authors:** Joseph Price

**Affiliations:** Philadelphia, Pa., 241 North 18th Street.


### DIAGNOSIS AND SURGICAL TREATMENT OF SUPPURATIVE PELVIC DISEASES.[1]

By JOSEPH PRICE, M. D., Philadelphia, Pa.

I ALWAYS feel highly complimented when asked to read a paper or discuss a subject before a society in good standing in the American Medical Association,—our great national, representative organisation,—to men with the passport of the national association to good professional society. I infer from my card that I am here to discuss two subjects: First, the diagnosis of suppurative forms of pelvic disease; second, the surgery of the same.

It will not be disputed that in dealing with the numerous diseases of women the element of the possible always enters in, that it is possible that something else—very much else—may exist other than that which we diagnosticate; that it is of the utmost importance that the highest faculty of diagnosis should be exercised—one educated by experience. I always urge upon my pupils the value of months, even years of service in some gynecological dispensary, the importance of methodical examination of patients, their examination before and after purgation. And further, while daily opportunity is afforded to examine patients suffering from a variety of troubles, they are to read both old and modern authorities on diagnosis and methods of examination. Had our profession that accurate knowledge of diagnosis they should possess, and which is, with the facilities at our command, of possible attainment, we would have but few chronic cases, and rarely ever a death from delayed or neglected operations. If an early operation follows a positive diagnosis early in the natural history of cystoma, fibroids, ectopic pregnancy, appendicitis or suppurating tubes, the results are about always perfect. The importance of early surgical interference in all forms of acute infectious diseases is vital if we would save all sufferers.

Our knowledge of pelvic inflammation or infectious troubles is now of a more precise nature than ever before. The confusing old nomenclature is fortunately being dropped or forgotten. Old authorities and practitioners failed to recognise small pelvic growths and tumors no larger than the uterus; they also failed to recognise tubal occlusion with retention of blood, pus or water—tubes quite as large in some cases as bananas or sweet potatoes. The stupid old practice of feeling the cervix and passing a sound is about as

1. Read before the Buffalo Academy of Medicine, June 28, 1898.

far as they went. Our examinations now are careful, methodical, our reasoning as to conditions more logical, our conclusions more in line with scientific principles; we differentiate between those conditions we can relieve or cure and those beyond our skill.

At present most clinicians make an earnest effort to determine early whether salpingitis with occlusion and adhesions exist or do not exist. In the early stages of salpingitis the symptoms are usually sufficiently prominent for a diagnosis. We have the history, the subjective signs, vaginitis, the discharge, local pain, temperature; objective symptoms, localised or general tenderness, fixation of uterus, tubes and ovaries—commonly more marked on one side than on the other—tortuous and enlarged tubes on one or both sides, the uterus commonly displaced and partially or completely fixed. In the more chronic forms of tubal and ovarian disease the tubes are usually found large, firm and tortuous, turned upon themselves on one or both sides of the uterus posteriorly and fixed. Ovarian abscesses are always perfectly round and symmetrical, exceedingly tender and painful. I would urge the importance of recognising tubal enlargement and fixation, and the relations distended tubes bear to a free or fixed uterus. The size, position, mobility and relation of uterus to surrounding parts is easily determined in most cases. In some cases it is very difficult to determine the position of the uterus, either by the bowel, vagina or an open abdominal incision. In a good number of cases you find the pelvic cavity full, nearly or quite, to the umbilicus. Beginning from above we find a fixed omentum, sometimes greatly thickened and disorganised; below the omentum we find the small bowel and all surrounding parts, sometimes adherent to small bowel involving sigmoid, head of cecum and appendix. The bladder is occasionally found adherent to omentum or bowel. In about 6 per cent. of suppurating appendages the appendix is involved on the right side. In about the same percentage the sigmoid is involved on the left side, is cheesy and disorganised to or through the mucous coat. Ideal, completed, perfect surgery is not easy. Patience, courage, endurance, a long surgical discipline and experience are essential to good and successful work. The operator should be as deft with his needle as the most skilled needlewoman.

Does this fact explain why so many operators at home and abroad practise the ancient method of vaginal puncture for tubal and ovarian suppurations? There has been a real back-out from what I would call complete surgery.

A few years ago I said to my friend Dr. McMurtry, of Louisville, that pelvic surgery as we were then doing it would become a lost art, that the profession was drifting that way and fast. I knew perfectly well that the men who were operating for pain were not prepared to deal with the complicated cases that would drift into their hands.

As yet we have no very precise knowledge of the particular germ responsible for the destruction of the pelvic viscera of so many women. We do know that if we could get rid of gonorrhea that pelvic suppurations would be rare. To say that neglected abortions or unscientific midwifery is responsible is putting it too strong and against facts and conditions of our everyday observation. I find the diagnosis of pelvic suppurations easy. I say easy because errors rarely occur. I see patients in my hospital and outside and in consultation with a good number of good clinicians. They give me a simple history of pelvic peritonitis, acute—the first or second attack—they also tell me they find fixation on the right, left or both sides, the uterus displaced, or in good position, and commonly a history of gonorrhea. Practitioners very commonly question husbands very pointedly on this subject. Years ago I did so; now I do not. I have come to regard it as a cruel practice; it avails nothing, the mischief has been done. I am satisfied without asking questions where the cause of the trouble lies. A study of pelvic disease by all our well-known methods from below, followed by careful study through a suprapubic opening, gives one a very perfect mental picture of both sides of the trouble. We are familiar with the pain and tenderness, with the tortuous, fixed appendages, or with the large ovarian abscesses, or the still larger fibroid, suppurating dermoids and large boggy blood accumulations. In the upper view we have the adherent omentum and bowel and large, smooth, tortuous and fixed tubes, filling the pelvic basin. If the pavilion extremity of the tube is fixed to the ovary we have the ovarian abscess, or tubo-ovarian puriform accumulations. If disorganisation or leakage of either tube or ovary has taken place, we have two, three or four or more pus accumulations within the fifth, walled in by adherent bowel and omentum above.

Could I dismiss or pass by all the adhesions, omental and bowel, bladder and uterine, disorganising appendix and sigmoid, numerous small and extensive adhesions of ileum, I would reject the abdominal and adopt the vaginal route, and rejoice that I had but little to do but the simple extirpation of the uterus and diseased appendages, as practised by one group of operators, or that of another, the least dangerous of all primary procedures, simple vaginal puncture and drainage. I say unhesitatingly that no one is prepared to treat these conditions without a good mental picture of them from both above and below.

Bowel lesions have always interested me and I am very careful in repairing them after the removal of suppurating tubes. I have always considered it a very important part of the work.

241 NORTH 18TH STREET.

